# Patients’ desires for anxiolytic premedication – an observational study in adults undergoing elective surgery

**DOI:** 10.1186/s12888-022-03845-y

**Published:** 2022-03-17

**Authors:** Frank Euteneuer, Stefan Kampmann, Stephen Rienmüller, Stefan Salzmann, Dirk Rüsch

**Affiliations:** 1 Department of Clinical Psychology and Psychotherapy, Philipps-University Marburg, Gutenbergstrasse 18, 35032 Marburg, Germany; 2 Department of Psychology, Clinical Psychology and Psychotherapy, Medical School Berlin, Calandrellistrasse 1-9, 12247 Berlin, Germany; 3 Philipps-University Marburg, Biegenstraße 10, 35037 Marburg, Germany; 4 University Hospital Giessen-Marburg (Marburg Campus), Department of Anesthesia and Intensive Care, Baldingerstrasse, 35043 Marburg, Germany

**Keywords:** Cross-sectional study, Preoperative anxiety, APAIS, Desire for premedication, Timing of premedication, Anxiolytic medication

## Abstract

**Background:**

Most patients experiencing preoperative anxiety would welcome support in coping with their anxiety. Anxiolytic medication is a common way to address preoperative anxiety. However, the proportion of patients who welcome anxiolytic medication preoperatively and the preferred time of taking it have not been studied thoroughly.

**Methods:**

Adult patients (*n* = 1000) scheduled to undergo elective surgery under general anesthesia were eligible to participate in this single-center observational study. Primary outcomes were the ratio of patients desiring anxiolytic medication (no/yes/on request) and the preferred time of taking it (evening before surgery/morning of day of surgery/on call to the operating room). Secondary outcomes included associations between different measures of anxiety (i.e., anxiety level according to the Amsterdam Preoperative Anxiety and Information Scale (APAIS) and anxiety status (no/yes)) and desire for anxiolytic medication (no/yes/on request). Primary outcomes were analyzed descriptively, and secondary outcomes were analyzed using multinomial logistic regressions.

**Results:**

Three hundred fifty-six (35.6%) out of 1000 patients desired anxiolytic medication and 239 (23.9%) patients would welcome anxiolytic medication on request. In patients reporting anxiety (no/yes; *n* = 493), 228 (46.2%) stated a clear desire for anxiolytic medication (yes) and 142 (28.8%) considered anxiolytic medication (on request). Patients’ preferences concerning the timing of premedication vary widely. In patients reporting a clear desire for anxiolytic medication (*n* = 356), the “morning of the day of surgery” was most frequently (*n* = 111, 31.2%) stated as the preferred time to get anxiolytic medication, followed by “on call to the operating room” (*n* = 51, 14.3%). All anxiety measures were significantly associated with desire for anxiolytic medication (*p* < 0.05).

**Conclusions:**

Given the importance of preoperative anxiety to patients, patients’ desire for anxiolytic medication should be considered when discussing the pros and cons of premedication. Individualized instead of standardized prescription and timing of premedication is recommended.

**Trial registration:**

German Clinical Trials Register (DRKS 00013319, approved 23/11/2017).

**Supplementary Information:**

The online version contains supplementary material available at 10.1186/s12888-022-03845-y.

## Background

Most patients undergoing elective surgery under general anesthesia experience anxiety [1–4]. Preoperative anxiety is associated with a negative emotional impact and a desire for support [5]. Accordingly, anxiety has been cited most frequently as the worst aspect of the perioperative period in a survey carried out in more than 16,000 patients [6]. A common way to help patients cope with preoperative anxiety is to administer anxiolytic medication, most commonly benzodiazepines [7–10]. However, there is inconsistent data regarding the anxiolytic effectiveness of benzodiazepines [11–19]. In addition, potentially detrimental side effects associated with their use including oversedation [10, 14] and postoperative neurocognitive deficits [14, 20] raise doubts concerning the benefits of benzodiazepines for premedication in particular for elderly people [21]. In the context of discussing the pros and cons of anxiolytic premedication in general (and not of a specific class of drugs used most commonly for this purpose), patient’s desire for anxiolytic premedication should be considered. Surprisingly, this important aspect has not been examined thoroughly so far despite the high importance of preoperative anxiety to patients [5, 6]. Furthermore, little is known regarding the preferred time of taking anxiolytic medication during the preoperative period. In addition, even though it can be assumed that increasing levels of anxiety are linked to an increasing desire for anxiolytic preoperative medication (premedication), associations between intensity of anxiety and need for premedication have never been examined. Accordingly, the impact of patient variables that have been shown to be associated with preoperative anxiety (e.g. female gender [22–25]) on associations between anxiety level and desire for premedication have not been studied either. It is also unknown if the difference between intensities of anesthesia anxiety and surgery anxiety [4, 23] is reflected in patient’s desire for premedication. Even though many studies have reported on intensities of preoperative anxieties [4, 23, 26], it is also unclear whether measurement of intensity of preoperative anxiety and the division of patients into those with high and low anxiety as suggested by Moerman and colleagues [22] on a routine basis makes clinically sense to identify patients that desire premedication.

This study is part of a large cross-sectional questionnaire survey investigating various issues related to preoperative anxiety. The survey’s results with regard to associations between preoperative anxiety and resulting emotional distress and desire for support in coping with anxiety have been published very recently [5]. This paper focuses on important aspects of patient’s desire for anxiolytic medication prior to elective surgery and extends previous research by primarily examining:

- the ratio of patients who desire preoperative anxiolytic medication
- what time patients pre﻿fer to take premedication.

Secondary aims were:

c) to study how patient’s desires for premedication relate to different measures of preoperative anxiety (i.e., anxiety levels of different anxiety dimensions (e.g. anesthesia anxiety) and anxiety status (self-classification as being anxious vs. non-anxious)).
d) to examine the accuracy of anxiety scores in general and the anxiety score suggested to identify patients with high anxiety in particular to predict a desire for anxiolytic premedication.

## Methods

This single-center observational study was conducted at Marburg University Hospital from November 2017 to October 2018. The survey was approved by the local ethics committee (Ethics committee of the Medical faculty of Marburg University, approval number 134/17, dated 10/10/2017) and registered with the German Clinical Trials Register (DRKS 00013319, approved 23/11/2017).

### Inclusion criteria

Patients 18 years or older scheduled to undergo non-emergency surgery under general anesthesia.

### Exclusion criteria

Patients scheduled to undergo procedures that could be performed under regional anesthesia only were not eligible. Other exclusion criteria were: illiteracy, insufficient knowledge of the German language, and visual impairments resulting in an inability to complete the questionnaire.

### Informed consent

In accordance with the decision of the local ethics committee, no written consent for participation was required because of the voluntary character of this anonymous survey. After information was provided to eligible patients about the methodology and aims of the survey, informed consent to participate was taken verbally, followed by completion of the questionnaire. Completion of the questionnaire could be stopped at any time without giving any reason.

### Data collection

Recruitment of participants took place at the pre-anesthetic evaluation clinic of Marburg University Hospital in patients waiting for their preoperative face-to-face interview with a physician of the Department of Anesthesia and Intensive Care. Patients who accepted the invitation to take part in the survey completed the questionnaire before the interview. Completion took on average about 10 minutes. A member of the study team was present while subjects completed the questionnaire to answer questions patients might have had.

### Questionnaire

The questionnaire consisted of the following seven parts:

Part 1 asked for sociodemographic variables, the scheduled procedure, the surgical consent, and previous surgeries.

Part 2 assessed anxiety about surgery and/or anesthesia (no/yes). Patients who reported anxiety were requested to answer a) whether the reported anxiety was related to surgery, anesthesia, or both, b) whether the anxiety was perceived as emotionally distressful or as unsettling (no/yes), and c) whether they would welcome to receive any kind of assistance or support from anesthesiologists in coping with their anxiety (no/yes).

Part 3 contained two numeric rating scales (NRS) ranging from 0 (no anxiety) to 10 (extreme anxiety) to measure anxiety related to anesthesia and surgery separately.

Part 4 contained a German version ([27], Additional file 1 – Supplementary Table 1) of the Amsterdam Preoperative Anxiety and Information Scale (APAIS [22], Additional file 1 – Supplementary Table 2). The APAIS contains six items (statements) to assess the level of patients’ anxiety about anesthesia (APAIS-A-An, two items), their anxiety about surgery (APAIS-A-Su, two items), and their need for information concerning anesthesia (APAIS-I-An, one item) and surgery (APAIS-I-Su, one item). Total preoperative anxiety (APAIS-A-T, four items) is the sum of anxiety about anesthesia (APAIS-A-An) and anxiety about surgery (APAIS-A-Su). Participants were asked to indicate the statements applicability on a 1 (not at all) to 5 (extremely) Likert scale. Items included in the different subscales and score ranges of subscales are summarized in Additional file 1 – Supplementary Table 3. The APAIS has been described in detail previously. Validated versions exist in numerous languages [22, 28–35] including German [27], and it can be considered as the most commonly used instrument to measure preoperative anxiety. Results of a previous survey in more than 3.000 patients showed that the reliability (Cronbach’s α) of the four anxiety items (“anxiety scale”) and the two information items (“information scale”) were 0.87 and 0.74, respectively [4].

Part 5 addressed those survey participants who reported to have anxiety (in part 2 of the questionnaire), how much they sensed this anxiety about anesthesia and surgery to be emotionally distressful or unsettling using numeric rating scales with a range from 0 (not emotionally distressful/unsettling) to 10 (extremely emotionally distressful/unsettling).

In part 6 patients were asked to write bullet points what their anxiety about surgery and/or anesthesia was based on. Anxiety provoking aspects were supposed to be listed in order of concern, starting with the most concerning aspect.

In part 7 patients were first informed that one option to alleviate anxiety is to take anxiety-reducing or calming drugs. Participants were then requested to report if they would like to have anxiolytic medication to alleviate their anxieties and worries (no/yes/on request). The following questions were supposed to be answered only by subjects who replied “yes” to the previous statement, i.e., those subjects who would clearly welcome to get anxiolytic medication. Patients were asked what time they would like to receive the anxiolytic medication (the evening before surgery (in short: evening)/in the morning or during the course of the morning of the day of surgery to bridge the waiting period until called to the operating room (in short: morning)/on call to the operating room (in short: on call); multiple answers possible) and if they would like to get any other kind of support in addition to the anxiolytic medication (no/yes). Patients who answered “yes” to the previous question were requested to write bullet points how they could be best helped.

### Primary and secondary outcomes

The primary outcomes of this study were the fraction of patients who reported a desire for anxiolytic medication (study aim 1A) and the preferred time to take it (study aim 1B). Secondary outcomes were associations between different measures of preoperative anxiety (i.e., anxiety levels of different anxiety dimensions (anesthesia anxiety, surgery anxiety, and total anxiety comprising anesthesia and surgery anxiety) according to APAIS and NRS as well as anxiety status (self-classification as being anxious vs. non-anxious) according to the question on anxiety (no/yes)) and desire for anxiolytic medication (no/yes), (study aim 2A). These associations were further examined by analyzing APAIS anxiety scores (study aim 2B-1) and the established APAIS anxiety score used for categorization (patients with high vs. low anxiety [22]) in particular (study aim 2B-2) concerning their accuracy to predict a desire for preoperative anxiolytic medication.

### Sample size

The sample size calculated for the survey was based upon the requirements for another study [5] that was also part of this survey. For that study [5] which focused on assessing the associations between preoperative anxiety and perceived emotional distress as well as desire for support, we calculated a sample size of 1000 patients since this would allow detecting even small correlations between anxiety and perceived emotional distress.

### Statistical analyses

Procedures were graded depending on their extent and invasiveness as “minor”, “intermediate” and “major” (Additional file 2 – Supplementary Table 4), similar to the classification previously published by Caumo and colleagues [36].

Descriptive statistics were calculated for all study variables and all results (part seven of the questionnaire) relevant to assess the ratio of patients who desire preoperative anxiolytic medication (study aim 1A) and the proportion of patients preferring the different time points for premedication (study aim 1B) . Associations between anxiety status and preferred time of premedication were examined using chi square tests in addition to explore study aim 1B. Column wise comparisons were carried out to test for specific differences in proportions between patients with anxiety and those with no anxiety for each time category.

Multinomial logistic regressions were calculated to examine associations between continuous (APAIS, NRS) and categorical (“anxiety status” according to the question in part 2 of the questionnaire on anxiety: no/yes, i.e., self-classification as being non-anxious vs. anxious) measures of preoperative anxiety and desire for anxiolytic medication (study aim 2A). These analyses were conducted for all patients and subgroups with different anxiety statuses (non-anxious vs. anxious). In terms of APAIS and NRS subscales (i.e., anesthesia and surgery anxiety score), additional multivariate models were run to examine whether each anxiety dimension has unique associations with the desire for anxiolytic medication when considering both subscales simultaneously. All regressions were rerun adjusting for variables that may theoretically be related with anxiety and/or the desire for anxiolytic premedication to examine whether measures of anxiety have unique associations with the desire for anxiolytic medication. These adjusted models include age, gender, education, number of previous surgeries, and grade of procedure [2, 22, 23, 25, 26, 36–38].

In addition, exploratory moderator analyses were calculated using logistic regression testing for the interaction between gender and preoperative anxiety scores (APAIS) for the outcome “desire for anxiolytic medication” (no/yes/on request).

Receiver operating characteristic (ROC) curves were calculated based on APAIS-A-T scores to examine which anxiety (APAIS-A-T) cut-off score is most accurate to detect patients who would welcome anxiolytic medication (study aim 2B1). This procedure was also applied to examine in particular the utility of applying an APAIS-A-T score >10 (suggested to identify patients with high anxiety [22]) to detect those who would welcome anxiolytic medication (yes vs. no and on request), (study aim 2B2). Youden index [39] was calculated as a measure of optimal cut-off scores when sensitivity and specificity are considered equally important.

Crosstabs were calculated to illustrate in detail the associations between the level of total preoperative anxiety (APAIS-A-T) and the desire for anxiolytic medication (no/yes/on request) in all patients and subgroups of patients depending on their anxiety status (non-anxious/anxious) (study aims 2A and 2B).

The sample included in this work has been used in a previous study [5]. However, none of the primary and secondary outcomes of the present study have been reported previously.

## Results

Recruitment of study participants started in November 2017 and stopped in October 2018 after 1000 patients had completed a questionnaire. During that time, 77 patients declined to participate in the survey, and five participants stopped completing the questionnaire. Patient characteristics are presented in Table 1.

**Table 1:** Sociodemographic variables and characteristics related to scheduled and previous surgeries of study participants

Variables	All patients (*n* = 1000)	Anxiety (*n* = 493)	No Anxiety (*n* = 507)
Age (years), [M (SD)]	56 (18.0)	56 (17.6)	57 (18.5)
Female [n (%)]	537 (53.9)	318 (31.9)	219 (22.0)
Male [n (%)]	459 (46.1)	171 (17.2)	288 (28.9)
Secondary school education [n (%)]			
Lower secondary degree	365 (36.8)	177 (17.8)	188 (18.9)
Medium secondary degree	319 (32.1)	155 (15.6)	164 (16.5)
Upper secondary degree	293 (29.5)	151 (15.2)	142 (14.3)
Without secondary school degree	16 (1.6)	5 (0.5)	11 (1.1)
Number of previous surgeries [n (%)]			
None	86 (8.6)	53 (10.8)	33 (6.5)
1 – 2	312 (31.3)	163 (33.1)	149 (29.4)
> 2	600 (60.1)	276 (56.0)	324 (63.9)
Time of intervention [n (%)]			
Same day	29 (3.0)	17 (3.4)	12 (2.4)
Following day	285 (29.1)	145 (29.4)	140 (27.6)
Later than following day	665 (67.9)	321 (65.1)	344 (67.9)
Consent for procedure ^a^ [n (%)]			
No	270 (27.2)	131 (26.6)	139 (27.4)
Yes	722 (72.8)	358 (72.6)	364 (71.8)
Grade of procedure ^b^ [n (%)]			
Minor	571 (59.9)	264 (53.5)	307 (60.6)
Intermediate	228 (23.9)	116 (23.5)	112 (22.1)
Major	155 (16.2)	90 (18.3)	65 (12.8)
Surgical discipline [n (%)]			
Ophthalmic	181 (18.2)	85 (17.2)	96 (18.9)
Gynecological	165 (16.6)	98 (19.9)	67 (13.2)
Ears, nose and throat	160 (16.2)	66 (13.4)	94 (18.5)
General	125 (12.6)	66 (13.4)	59 (11.6)
Urological	99 (10.0)	40 (8.1)	59 (11.6)
Neurosurgical	77 (7.8)	43 (8.7)	34 (6.7)
Oral and maxillofacial	69 (7.0)	28 (5.7)	41 (8.1)
Cardiac	55 (5.5)	29 (5.9)	26 (5.1)
Orthopedic	33 (3.3)	18 (3.7)	15 (3.0)
Trauma	16 (1.6)	9 (1.8)	7 (1.4)
Dermatological	12 (1.2)	5 (1.0)	7 (1.4)

*Anxiety* patients who reported anxiety (no/yes), *No anxiety* patients who did not report anxiety (no/yes). ^a^ completion of the questionnaire took place after surgical preoperative assessment and consent for surgical procedure (no/yes). ^b^ the assignment of the different procedures to the three grades of surgeries is shown in Additional file 2.

### Anxiety

Approximately half of the subjects (49.3%) reported anxiety (no/yes). Among these, about two-thirds perceived their anxiety as emotionally distressful and/or unsettling (no/yes) [5]. Mean anxiety scores according to APAIS and NRS are presented in Table 2.

**Table 2:** APAIS and NRS anxiety scores

Scale	All patients [M (SD)]	No anxiety [M (SD)]	Anxiety [M (SD)]
APAIS-A-Su (range 2 -10)	5.1 (2.3)	3.7 (1.6)	6.5 (2.0)
APAIS-A-An (range 2 -10)	4.1 (2.0)	3.0 (1.1)	5.2 (2.0)
APAIS-A-T (range 4 -20)	9.2 (3.8)	6.7 (2.4)	11.7 (3.2)
NRS-A-Su (range 0-10)	3.6 (2.9)	1.5 (1.7)	5.6 (2.5)
NRS-A-An (range 0-10)	2.9 (2.7)	1.1 (1.4)	4.6 (2.6)
NRS-A-T (range 4-20)	6.4 (5.2)	2.7 (2.9)	10.3 (4.2)

*APAIS* Amsterdam preoperative anxiety and information scale, *NRS* Numeric rating scale, *APAIS-A-Su* APAIS anxiety about surgery score, *APAIS-A-An* APAIS anxiety about anesthesia score, *APAIS-A-T* APAIS anxiety about anesthesia and surgery score (total APAIS anxiety score), *NRS-A-Su* NRS anxiety about surgery score *NRS-A-An* NRS anxiety about anesthesia score *NRS-A-T* NRS anxiety about anesthesia and surgery score (total NRS anxiety score), *No anxiety* Patients who did not report anxiety (no/yes), *Anxiety* Patients who reported anxiety (no/yes).

### Desire for preoperative anxiolytic medication

About one-third of all patients reported a clear desire (yes) to receive anxiolytic medication, and about a quarter of patients considered (on request) taking premedication (no/yes/on request). Less than 40% of patients stated no need for anxiolytic medication (no/yes/on request). In patients who reported anxiety (no/yes), three quarters expressed either a clear desire (yes) to get anxiolytic medication or considered (on request) taking anxiolytic medication and less than a quarter reported no desire to receive anxiolytic medication (no/yes/on request). Results concerning the desire for anxiolytic medication are summarized in Table 3.

**Table 3:** Desire for anxiolytic medication

Desire for anxiolytic medication	Yes [n (%)]	On request [n (%)]	No [n (%)]	Not reported [n (%)]
All patients (*n* = 1000)	356 (35.6)	239 (23.9)	389 (38.9)	16 (1.6)
Anxiety (*n* = 493)	228 (46.2)	142 (28.8)	114 (23.1)	9 (1.8)
No anxiety(*n* = 507)	128 (25.2)	97 (19.1)	275 (54.2)	7 (1.4)

*Anxiety* Patients who reported anxiety (no/yes), *No anxiety* Patients who did not report anxiety (no/yes).

### Preferred time of premedication

Patients’ preferences concerning the timing of premedication vary widely. The most frequently reported time was “the morning / the course of the morning of the day of surgery until called to the operating room” followed by “on call to the operating room” comprising about half of patients with a clear desire for anxiolytic premedication.

The association between anxiety status (anxious vs. non-anxious) and preferred time of premedication was significant (*p* = .022). Column wise comparisons indicated that the proportion of patients with anxiety was lower than the proportion of patients without anxiety among those who prefer medication on call (*p* = < 0.05). All results concerning the preferred time points of premedication are presented in Table 4.

**Table 4:** Preferred time of premedication

	All patients (*n* = 356)	Anxiety (*n* = 228)	No anxiety (*n* = 128)
Morning	111 (31.2)	75 (32.9)	36 (28.1)
On call	69 (19.4)	33 (14.5) ^a^	36 (28.1) ^a^
Evening and morning	51 (14.3)	35 (15.4)	16 (12.5)
Evening	48 (13.5)	30 (13.2)	18 (14.1)
Evening, morning and on call	32 (9.0)	26 (11.4)	6 (4.7)
Morning and on call	29 (8.1)	21 (9.2)	8 (6.3)
Evening and on call	15 (4.2)	8 (3.5)	7 (5.5)
Missing data	1 (0.3)	0 (0.0)	1 (0.8)

*Anxiety* Patients who reported anxiety (no/yes), *No anxiety* Patients who did not report anxiety (no/yes), *Morning* In the morning or during the course of the morning of the day of surgery to bridge the waiting period until called to the operating room, *Evening* The evening before surgery, *On call* On call to the operating room. ^a^ *p* < 0.05 for column wise comparison.

### Associations between anxiety and desire for anxiolytic medication

Results from multinomial logistic regression analyses are presented in Table 5. We report unstandardized estimates and odds ratios for associations between measures of preoperative anxiety and the desire for anxiolytic medication for all patients and subgroups depending on their anxiety status (anxiety: no/yes). When looking at both categories of anxiety status (anxiety: no/yes), patients who reported anxiety (no/yes) were 4.30 times more likely to desire anxiolytic medication (yes vs. no) and 3.53 times more likely to desire anxiolytic medication on request (on request vs. no), which is an increase by 330% and 253%, respectively. In bivariate multinomial logistic regression models, all continuous measures of anxiety were significantly associated with the desire for anxiolytic medication (yes vs. no, *p*s = < 0.001–0.004; on request vs. no, *p*s = < 0.001–0.013). For example, with an increase of one score in APAIS total anxiety, the likelihood to desire anxiolytic medication (yes vs. no) increased by 28% in the whole sample. When considering both APAIS subscales simultaneously in regression analyses, both anesthesia anxiety and surgery anxiety had independent associations with the desire for anxiolytic medication in the whole sample. However, in patients who reported anxiety (no/yes), APAIS anesthesia (but not surgery) anxiety was significantly related to the desire for anxiolytic medication (yes vs. no), indicating that anesthesia anxiety has aspects that relate to the desire for anxiolytic medication in this subsample. A similar pattern was observed for the NRS subscales in terms of patients who reported anxiety. Results from adjusted regression models (Additional file 3 – Supplementary Table 5) were similar to those from unadjusted models (Table 5). Exploratory moderation analyses did not indicate that gender moderated associations between preoperative anxiety scores (APAIS) and the desire for anxiolytic medication.

**Table 5:** Associations between measures of preoperative anxiety and the desire for anxiolytic medication

Variable	Desire for anxiolytic medication					
	Yes ^a^			On request ^a^		
	B	*p*	OR [95 %CI]	B	*p*	OR [95 %CI]
All patients						
Self-reported anxiety (no vs. yes)	1.46	< 0.001	4.30 [3.16, 5.84]	1.26	< 0.001	3.53 [2.52, 4.95]
APAIS total anxiety score	0.25	< 0.001	1.28 [1.22, 1.34]	0.20	< 0.001	1.22 [1.16, 1.29]
APAIS anxiety dimensions						
Bivariate						
APAIS anesthesia anxiety score	0.46	< 0.001	1.58 [1.44, 1.73]	0.39	< 0.001	1.47 [1.33, 1.63]
APAIS surgery anxiety score	0.33	< 0.001	1.40 [1.30, 1.50]	0.27	< 0.001	1.30 [1.21, 1.41]
Multivariate ^b^						
APAIS anesthesia anxiety score	0.33	< 0.001	1.39 [1.25, 1.54]	0.28	< 0.001	1.32 [1.18, 1.49]
APAIS surgery anxiety score	0.19	< 0.001	1.21 [1.11, 1.31]	0.14	0.002	1.15 [1.05, 1.27]
NRS total anxiety score	0.19	< 0.001	1.21 [1.17, 1.25]	0.16	< 0.001	1.18 [1.13, 1.22]
NRS anxiety dimensions						
NRS anesthesia anxiety score	0.34	< 0.001	1.41 [1.32, 1.50]	0.28	< 0.001	1.32 [1.23, 1.42]
NRS surgery anxiety score	0.30	< 0.001	1.35 [1.27, 1.43]	0.27	< 0.001	1.30 [1.22, 1.39]
Multivariate ^b^						
NRS anesthesia anxiety score	0.21	< 0.001	1.24 [1.14, 1.34]	0.14	< 0.001	1.15 [1.05, 1.27]
NRS surgery anxiety score	0.17	< 0.001	1.19 [1.10, 1.28]	0.18	< 0.001	1.20 [1.10, 1.30]
Patients who reported anxiety (no vs. yes)						
APAIS total anxiety score	0.17	< 0.001	1.19 [1.10, 1.28]	0.11	0.008	1.12 [1.03, 1.21]
APAIS anxiety dimensions						
Bivariate						
APAIS anesthesia anxiety score	0.28	< 0.001	1.32 [1.16, 1.50]	0.22	0.002	1.24 [1.08, 1.42]
APAIS surgery anxiety score	0.17	0.004	1.18 [1.06, 1.33]	0.07	0.237	1.08 [0.95, 1.22]
Multivariate ^b^						
APAIS anesthesia anxiety score	0.25	< 0.001	1.29 [1.13, 1.47]	0.21	0.004	1.23 [1.07, 1.42]
APAIS surgery anxiety score	0.10	0.086	1.11 [0.99, 1.25]	0.03	0.651	1.03 [0.91, 1.17]
NRS total anxiety score	0.14	< 0.001	1.12 [1.09, 1.23]	0.10	0.002	1.10 [1.04, 1.18]
NRS anxiety dimensions						
NRS anesthesia anxiety score	0.21	< 0.001	1.23 [1.12, 1.35]	0.13	0.011	1.14 [1.03, 1.26]
NRS surgery anxiety score	0.17	< 0.001	1.19 [1.08, 1.30]	0.13	0.013	1.14 [1.03, 1.26]
Multivariate ^b^						
NRS anesthesia anxiety score	0.17	0.001	1.19 [1.08, 1.31]	0.10	0.065	1.11 [0.99, 1.23]
NRS surgery anxiety score	0.11	0.027	1.12 [1.01, 1.24]	0.10	0.071	1.11 [0.99, 1.23]
Patients who reported no anxiety (no vs. yes)						
APAIS total anxiety score	0.21	< 0.001	1.23 [1.13, 1.35]	0.18	< 0.001	1.20 [1.08, 1.32]
APAIS anxiety dimensions						
Bivariate						
APAIS anesthesia anxiety score	0.43	< 0.001	1.53 [1.27, 1.86]	0.34	0.002	1.41 [1.14, 1.74]
APAIS surgery anxiety score	0.26	< 0.001	1.29 [1.13, 1.48]	0.24	0.002	1.27 [1.10, 1.48]
Multivariate ^b^						
APAIS anesthesia anxiety score	0.32	0.007	1.37 [1.09, 1.73]	0.21	0.119	1.23 [0.95, 1.59]
APAIS surgery anxiety score	0.14	0.109	1.15 [0.97, 1.36]	0.16	0.079	1.18 [0.98, 1.41]
NRS total anxiety score	0.19	< 0.001	1.21 [1.12, 1.31]	0.19	< 0.001	1.21 [1.12, 1.32]
NRS anxiety dimensions						
NRS anesthesia anxiety score	0.37	< 0.001	1.45 [1.24, 1.68]	0.35	< 0.001	1.41 [1.19, 1.68]
NRS surgery anxiety score	0.30	< 0.001	1.35 [1.18, 1.53]	0.32	< 0.001	1.37 [1.19, 1.58]
Multivariate ^b^						
NRS anesthesia anxiety score	0.22	0.057	1.25 [0.99, 1.57]	0.15	0.232	1.16 [0.91, 1.48]
NRS surgery anxiety score	0.17	0.085	1.18 [0.98, 1.42]	0.23	0.024	1.26 [1.03, 1.53]

*APAIS* Amsterdam preoperative anxiety and information scale, *NRS* numeric rating scale. ^a^ Reference category: No desire for anxiolytic medication. ^b^ Models included both anxiety dimensions simultaneously to examine whether anesthesia anxiety and surgery anxiety have unique associations with the desire for anxiolytic medication.

### Prediction of desire for anxiolytic medication

The anxiety levels to predict most accurately a clear desire for anxiolytic medication (yes vs. no and on request) when equally weighting sensitivity and specificity were APAIS-A-T > 8 and APAIS-A-T > 9 given their identical Youden index of 0.235. These cut-off scores had a sensitivity of 0.669 and 0.601 and a specificity of 0.566 and 0.634, respectively (see Additional file 4 for ROC curve).

In comparison, the anxiety level commonly used to detect patients with high anxiety (“anxiety cases”, APAIS-A-T score > 10) was less accurate in predicting a desire for anxiolytic medication when equally weighting sensitivity and specificity (Youden index of 0.188 with a sensitivity of 0.462 and a specificity of 0.726).

There was no lower anxiety level threshold concerning a desire for anxiolytic medication. Even the lowest levels of total preoperative anxiety (APAIS-A-T = 5 or 6) were associated with a desire for anxiolytic medication in some patients. Details concerning the need for anxiolytic medication depending on total preoperative anxiety (APAIS-A-T) are presented using crosstabs (Additional files 5, 6 and 7).

## Discussion

Results of this study carried out in 1000 patients scheduled for elective surgery contain novel information about various aspects related to patients’ desire for anxiolytic medication and the associations between preoperative anxiety and patients’ need for anxiolytic medication.

### Desire for anxiolytic medication

The main result of this study is that about one-third of our study sample expressed an explicit desire for anxiolytic medication, and about a quarter of patients considered anxiolytic medication. To our knowledge, this is the first study reporting in detail on the number (fraction) of adult patients who explicitly desire, who consider, and who do not want anxiolytic medication when undergoing elective surgery. In a previous randomized controlled multicenter trial studying primarily the effects of sedative premedication, participants were also asked whether they would like to receive a hypnotic the night before surgery and/or a premedication before surgery [19]. The fraction of patients requesting premedication was reported to range from 56 – 62% in the three study groups without being precise at what time exactly on the day of surgery patients were offered to get the premedication. Despite the many differences in study designs, the results of their study are consistent with the results of this study, demonstrating that a large fraction of patients undergoing elective surgery requests anxiolytic medication preoperatively.

This study adds important information concerning the discussion about the pros and cons of sedative and anxiolytic premedication. On the one hand, the results of this study underline the importance of anxiolytic medication to adult patients undergoing elective surgery. On the other hand, there is a controversial discussion about the use of benzodiazepines that have been administered most frequently for more than three decades as premedication to alleviate anxiety and to provide sedation [7–10]. An increasing reluctance to prescribe benzodiazepines for premedication, especially in elderly patients, is based on inconsistent results regarding their anxiolytic effectiveness [11–18] and on potentially detrimental side effects including oversedation [10, 14] and postoperative neurocognitive deficits [14, 20]. The above aspects highlight the need to find alternative anxiolytic drugs for patients who would welcome anxiolytic medication, but in whom the administration of benzodiazepines is associated with the risk of side effects.

Subgroup analysis according to patients’ self-classification as being anxious or non-anxious also revealed clinically important results. Most importantly, it showed that the rate of desire for premedication is higher in the subgroup of anxious patients suggesting an association between the two variables which is confirmed by results of the logistic regression analyses of this study.

### Timing of anxiolytic medication

The second main result of this study is that patients’ preferences concerning the timing of premedication were highly variable. This variability was observed in all patients and both subgroups (anxious and non-anxious patients). Therefore, any standardized prescription of premedication does not adequately reflect patients' wishes regarding the timing of premedication. Instead, an individualized prescription according to patients’ preferences is recommended provided this is consistent with patient safety. These results and conclusions are not surprising to us. However, despite an abundance of studies dealing with various aspects related to premedication, we are not aware of any study that has examined in detail the timing of premedication from the patients’ point of view. Therefore, we believe that the findings of this study can add helpful information in the context of patient care during the preoperative period.

### Associations between anxiety and desire for anxiolytic medication

To our knowledge, associations between anxiety and desire for anxiolytic medication have not been studied yet. Therefore, the following results are novel, though not unexpected. First, patients explicitly reporting anxiety were 4.3 more likely to desire and 3.53 times more likely to consider anxiolytic premedication. Second, both APAIS and NRS anxiety scores were significantly associated with a desire for anxiolytic medication. Both anesthesia and surgery anxiety had unique associations with the desire for anxiolytic medication in all patients. Remarkably, in patients explicitly reporting anxiety, only anesthesia anxiety was significantly associated with the desire for anxiolytic medication when considering both anxiety dimensions simultaneously. These results are somewhat surprising in the light of the numerous studies that have examined intensities of anesthesia and surgery anxiety. The overwhelming majority of these studies (e.g .[3, 4, 23, 24, 40, 41]) have demonstrated that average surgery anxiety levels are significantly higher than average anesthesia anxiety levels suggesting that surgery anxiety could have a stronger impact on the desire for anxiolytic medication. Results of this study do not confirm this assumption and emphasize the importance of anesthesia anxiety concerning the desire for anxiolytic medication. Contrary to our assumption based on previous findings that female gender is the strongest risk factor for preoperative anxiety [25] this study could not confirm an impact of gender on associations between anxiety level and desire for premedication.

### Prediction of desire for anxiolytic medication

ROC analyses revealed that a total anxiety level (APAIS-A-T) > 8 and > 9 best predicted a desire for anxiolytic medication in all patients of this study. Considering the low sensitivity of these scores, their use in everyday clinical practice to determine what patients need anxiolytic premedication does not make sense.

Accordingly, the use of an APAIS-A-T score > 10 as suggested by Moerman and colleagues to identify patients with high anxiety is not helpful either to predict which patients need anxiolytic medication. Considering the limited benefit of identifying patients with high anxiety to determine which patients perceive their anxiety as emotionally distressful or unsettling and what patients desire support in coping with their anxiety [5], results of this study strongly suggest that the concept of dividing patients into those with high and low anxiety is questionable from a clinical point of view. In addition, considering that there was no lower anxiety level threshold for the desire for anxiolytic medication, we recommend asking all patients whether they would welcome premedication.

### Limitations

The generalizability of the results of this study is limited because of the single center design of this study. In addition, the present study focuses on very specific aspects related to preoperative anxiety and its treatment using anxiolytic medication, namely the proportion of patients who desire anxiolytic medication and the favored time to get these drugs as primary outcome. Therefore, many psychological aspects including the doctor-patient relationship and psychological variables that could mediate preoperative anxiety have not been examined concerning their impact on the primary outcomes. Likewise, the usefulness of possible psychological interventions offered to patients to cope with their preoperative anxiety has not been studied yet. Moreover, we did not record whether patients were day-case patients or in-patients even though this can be assumed to impact the desire for anxiolytic premedication.

## Conclusions

Results of this study emphasize the importance of anxiolytic medication to patients scheduled to undergo surgery to cope with preoperative anxiety. A general abandonment of anxiolytic medication because of the adverse side effects of one particular class of drugs is not consistent with the needs of many patients. Therefore, the results of this study indicate a need for developing anxiolytic drugs without or fewer side effects that limit their use in the preoperative period. The findings of this study also highlight that a standardized timing of the administration of premedication is not consistent with patients' needs. In summary, results of this study suggest that an individualized prescription and timing of premedication considering all perioperative medical aspects including patients’ desires seems most reasonable.

## Supplementary Information

**Additional file 1: Supplementary Table 1.** German version of the Amsterdam Preoperative Anxiety and Information Scale (APAIS). Description: Wording of the German translation of the English version of the APAIS published by Moerman and colleagues [22] and validated by Berth and colleagues [27]. **Supplementary Table 2.** English version of the APAIS. Description: Wording of the English version of the APAIS published by Moerman and colleagues [22].
**Supplementary Table 3.** APAIS scale and APAIS subscales. Description: Overview of the different APAIS subscales, the items that define them, their score ranges, and their abbreviations used in the present paper.

**Additional file 2: Supplementary Table 4.** Grading of surgical procedures. Description: Allocation of all procedures included in the study to 3 different grades (minor, intermediate and major) depending on their extent and invasiveness, similar to the classification previously published by Caumo and colleagues [36].

**Additional file 3: Supplementary Table 5.** Adjusted associations. Description: Table showing associations between measures of preoperative anxiety and desire for anxiolytic medication after adjusting for potential covariates.

**Additional file 4: Supplementary Figure 1.** ROC curve. Description: Receiver operating characteristic (ROC) curve of prediction of desire for preoperative anxiolytic medication (premedication) using total anxiety scores.

**Additional file 5: Supplementary Table 6.** Crosstab of desire for anxiolytic medication (no/yes/on request) depending on total anesthesia and surgery anxiety (APAIS-A-T) level in all patients.

**Additional file 6: Supplementary Table 7.** Crosstab of desire for anxiolytic medication (no/yes/on request) depending on total anesthesia and surgery anxiety (APAIS-A-T) level in anxious patients (no/yes).

**Additional file 7: Supplementary Table 8**. Crosstab of desire for anxiolytic medication (no/yes/on request) depending on total anesthesia and surgery anxiety (APAIS-A-T) level in non-anxious patients (no/yes).

## Data Availability

The datasets used and analyzed during the current study are available from the corresponding author on reasonable request.

## Abbreviations

APAIS: Amsterdam preoperative anxiety and information scale
APAIS-A-An: APAIS anesthesia anxiety score
APAIS-A-Su: APAIS surgery anxiety score
APAIS-A-T: APAIS total anxiety score
APAIS-I-T: APAIS total information score
NRS: numeric rating scale
OR: odds ratio
ROC: receiver operating characteristic
SD: standard deviation
