# Supplementary material for: Patients’ desires for anxiolytic premedication – an observational study in adults undergoing elective surgery
Source: BMC Psychiatry. 2022 Mar 17;22:193. doi: 10.1186/s12888-022-03845-y (PMC8932104; doi:10.1186/s12888-022-03845-y)
Supplement: Supplementary file 1 — Additional file 1: Supplementary Table 1. German version of the Amsterdam Preoperative Anxiety and Information Scale (APAIS). Description: Wording of the German translation of the English version of the APAIS published by Moerman and colleagues [22] and validated by Berth and colleagues [27]. Supplementary Table 2. English version of the APAIS. Description: Wording of the English version of the APAIS published by Moerman and colleagues [22]. Supplementary Table 3. APAIS scale and APAIS subscales. Description: Overview of the different APAIS subscales, the items that define them, their score ranges, and their abbreviations used in the present paper. [file 12888_2022_3845_MOESM1_ESM.docx]

**Additional file 1** - APAIS

Supplementary Table 1 – APAIS in German (Part 4 of the questionnaire)

|  | **1**  (gar nicht) | **2**  (wenig) | **3**  (mittel) | **4**  (stark) | **5**  (extrem) |
| --- | --- | --- | --- | --- | --- |
| Ich mache mir Sorgen über die Anästhesie (Narkose) |  |  |  |  |  |
| Die Anästhesie (Narkose) geht mir ständig durch den Kopf |  |  |  |  |  |
| Ich möchte so viel wie möglich über die Anästhesie (Narkose) wissen |  |  |  |  |  |
| Ich mache mir Sorgen über die Operation |  |  |  |  |  |
| Die Operation geht mir ständig durch den Kopf |  |  |  |  |  |
| Ich möchte so viel wie möglich über die Operation wissen |  |  |  |  |  |

Supplementary Table 2 – APAIS in English (as presented in [22]).


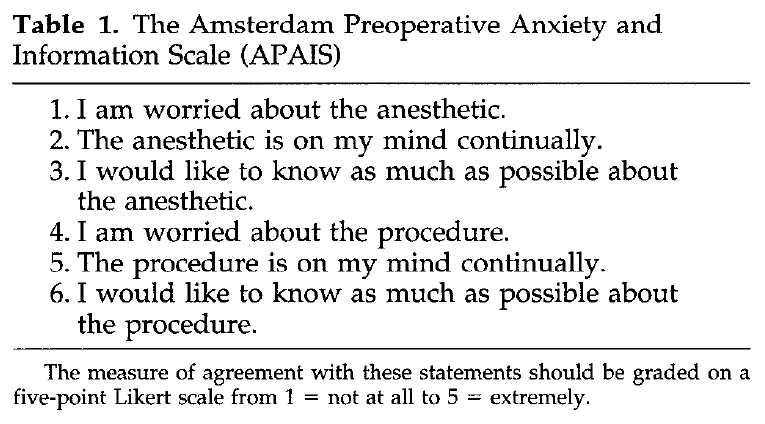


Supplementary Table 3 - APAIS scale and APAIS subscales

| APAIS (sub)scale | Items included | Score | Meaning |
| --- | --- | --- | --- |
| APAIS-T | 1 - 6 | 6 - 30 | Total anxiety and need for information |
| APAIS-A-An | 1 + 2 | 2 - 10 | Total anesthesia anxiety |
| APAIS-A-Su | 4 + 5 | 2 – 10 | Total surgery anxiety |
| APAIS-A-T | 1 + 2 + 4 + 5 | 4 – 20 | Total anesthesia and surgery anxiety |
