# Supplementary material for: Patients’ desires for anxiolytic premedication – an observational study in adults undergoing elective surgery
Source: BMC Psychiatry. 2022 Mar 17;22:193. doi: 10.1186/s12888-022-03845-y (PMC8932104; doi:10.1186/s12888-022-03845-y)
Supplement: Supplementary file 2 — Additional file 2: Supplementary Table 4. Grading of surgical procedures. Description: Allocation of all procedures included in the study to 3 different grades (minor, intermediate and major) depending on their extent and invasiveness, similar to the classification previously published by Caumo and colleagues [36]. [file 12888_2022_3845_MOESM2_ESM.docx]

**Additional file 2 -** Grading of surgical procedures

Supplementary Table 4 - Grading of surgical procedures

| Grade | Surgical procedure |
| --- | --- |
| Minor | Abscess incision and drainage, arthroscopic shoulder surgery (e.g. subacromial decompression, shoulder ligament reconstruction), basal cell carcinoma surgery, breast conserving therapy, breast implants removal, bursal resection, cardiac pacemaker implantation, endobronchial ultrasound with/without transbronchial needle aspiration, excision of cutaneous melanoma and sentinel lymph nodes, hematoma drainage, herniotomy (e.g. inguinal and umbilical repair), kyphoplasty, laparoscopic procedures (appendectomy, cholecystectomy, adnexal surgery for benign diseases), lymphadenectomy (cervical, axillary, inguinal), minor osteosynthesis surgery (e.g. osteosynthesis for ac-joint separation), microlaryngoscopy with biopsy, ophthalmic surgery, pacemaker generator change, parotidectomy, removal of metal implants after fracture, scar revision surgery, septoplasty/septorhinoplasty, stapedectomy, surgery for pilonidal disease, thyroidectomy, tonsillectomy, tympanoplasty, ureterorenoscopy with/without placement of ureteral stent, vacuum assisted closure, wisdom tooth extraction. |

| Intermediate | Bimaxillary osteotomy, breast reduction surgery, carotid endarterectomy, cochlear implant implantation, hysterectomy (abdominal, vaginal), mastectomy, myomectomy, neck dissection, oophorectomy, radical prostatectomy, salpingo-oophorectomy, spinal disc surgery (e.g. lumbar discectomy), spinal surgery (e.g. laminectomy, spinal fusion), tuboplasty, ureterectomy, vulvectomy. |
| --- | --- |
| Major | Adrenalectomy, bile duct surgery, cardiac surgery with sternotomy, cholecystectomy (open), cystectomy, gastrectomy, hepatectomy (partial), hysterectomy (radical), intracranial surgical procedures, major joint surgery (e.g. revision total hip replacement), lung surgery with thoracotomy, nephrectomy, pancreatectomy, rectosigmoidectomy, splenectomy. |
