# Supplementary material for: Patients’ desires for anxiolytic premedication – an observational study in adults undergoing elective surgery
Source: BMC Psychiatry. 2022 Mar 17;22:193. doi: 10.1186/s12888-022-03845-y (PMC8932104; doi:10.1186/s12888-022-03845-y)
Supplement: Supplementary file 3 — Additional file 3: Supplementary Table 5. Adjusted associations. Description: Table showing associations between measures of preoperative anxiety and desire for anxiolytic medication after adjusting for potential covariates. [file 12888_2022_3845_MOESM3_ESM.docx]

**Additional file** 3 – Adjusted associations

Supplementary Table 5 - Associations between measures of preoperative anxiety and desire for anxiolytic medication after adjusting for potential covariates.

| Variable | Desire for anxiolytic medication ^a^ | | | | | | |
| --- | --- | --- | --- | --- | --- | --- | --- |
|  | Yes ^b^ | | |  | On request ^b^ | | |
|  | B | *p* | OR [95 %CI] |  | B | *p* | OR [95 %CI] |
| All patients  Self-reported anxiety (no vs. yes)  APAIS total anxiety score  APAIS anxiety dimensions  Bivariate  APAIS anesthesia anxiety score  APAIS surgery anxiety score  Multivariate ^c^  APAIS anesthesia anxiety score  APAIS surgery anxiety score  NRS total anxiety score  NRS anxiety dimensions  Bivariate  NRS anesthesia anxiety score  NRS surgery anxiety score  Multivariate ^c^  NRS anesthesia anxiety score  NRS surgery anxiety score  Patients who reported anxiety (no vs. yes)  APAIS total anxiety score  APAIS anxiety dimensions  Bivariate  APAIS anesthesia anxiety score  APAIS surgery anxiety score  Multivariate ^c^  APAIS anesthesia anxiety score  APAIS surgery anxiety score  NRS total anxiety score  NRS anxiety dimensions  Bivariate  NRS anesthesia anxiety score  NRS surgery anxiety score  Multivariate ^c^  NRS anesthesia anxiety score  NRS surgery anxiety score  Patients who reported no anxiety (no vs. yes)  APAIS total anxiety score  APAIS anxiety dimensions  Bivariate  APAIS anesthesia anxiety score  APAIS surgery anxiety score  Multivariate ^c^  APAIS anesthesia anxiety score  APAIS surgery anxiety score  NRS total anxiety score  NRS anxiety dimensions  Bivariate  NRS anesthesia anxiety score  NRS surgery anxiety score  Multivariate ^c^  NRS anesthesia anxiety score  NRS surgery anxiety score | 1.39  0.24  0.46  0.31  0.34  0.17  0.19  0.33  0.29  0.21  0.17  0.16  0.28  0.15  0.25  0.09  0.14  0.20  0.17  0.16  0.12  0.22  0.46  0.26  0.36  0.13  0.19  0.39  0.29  0.27  0.13 | < 0.001  < 0.001  < 0.001  < 0.001  < 0.001  < 0.001  < 0.001  < 0.001  < 0.001  < 0.001  < 0.001  < 0.001  < 0.001  0.016  < 0.001  0.175  < 0.001  < 0.001  0.001  0.002  0.026  < 0.001  < 0.001  < 0.001  0.005  0.138  < 0.001  < 0.001  < 0.001  0.032  0.192 | 4.02 [2.89, 5.58]  1.27 [1.21, 1.33]  1.58 [1.43, 1.74]  1.37 [1.27, 1.47]  1.40 [1.25, 1.58]  1.18 [1.08, 1.29]  1.20 [1.16, 1.25]  1.39 [1.30, 1.49]  1.33 [1.25, 1.42]  1.23 [1.13, 1.35]  1.18 [1.10, 1.28]  1.18 [1.08, 1.28]  1.32 [1.16, 1.50]  1.16 [1.03, 1.31]  1.29 [1.12, 1.48]  1.09 [0.96, 1.24]  1.15 [1.08, 1.23]  1.22 [1.10, 1.34]  1.19 [1.07, 1.31]  1.18 [1.06, 1.31]  1.13 [1.01, 1.26]  1.25 [1.13, 1.37]  1.59 [1.29, 1.95]  1.30 [1.13, 1.50]  1.43 [1.12, 1.83]  1.14 [0.96, 1.36]  1.21 [1.12, 1.32]  1.47 [1.24, 1.75]  1.34 [1.16, 1.54]  1.31 [1.02, 1.67]  1.14 [0.94, 1.39] |  | 1.21  0.20  0.39  0.25  0.31  0.12  0.16  0.28  0.26  0.15  0.17  0.10  0.21  0.04  0.21  -0.01  0.10  0.13  0.13  0.10  0.10  0.19  0.37  0.23  0.26  0.14  0.18  0.33  0.29  0.18  0.19 | < 0.001  < 0.001  < 0.001  < 0.001  < 0.001  0.014  < 0.001  < 0.001  < 0.001  0.002  < 0.001  0.034  0.005  0.509  0.007  0.992  0.004  0.019  0.020  0.080  0.080  0.001  0.001  0.004  0.056  0.154  < 0.001  < 0.001  < 0.001  0.171  0.078 | 3.36 [2.35, 4.82]  1.22 [1.16, 1.28]  1.48 [1.33, 1.65]  1.28 [1.18, 1.39]  1.36 [1.20, 1.54]  1.13 [1.03, 1.24]  1.17 [1.13, 1.22]  1.32 [1.22, 1.42]  1.29 [1.21, 1.38]  1.17 [1.06, 1.28]  1.18 [1.09, 1.29]  1.10 [1.01, 1.20]  1.24 [1.07, 1.43]  1.05 [0.92, 1.19]  1.23 [1.06, 1.43]  1.00 [0.87, 1.14]  1.11 [1.03, 1.18]  1.14 [1.02, 1.26]  1.14 [1.02, 1.26]  1.11 [0.99, 1.24]  1.11 [0.99, 1.24]  1.20 [1.08, 1.34]  1.45 [1.16, 1.82]  1.26 [1.08, 1.48]  1.30 [0.99, 1.70]  1.15 [0.95, 1.39]  1.20 [1.10, 1.32]  1.40 [1.16, 1.68]  1.34 [1.15, 1.56]  1.20 [0.93, 1.55]  1.21 [0.98, 1.48] |

*APAIS* Amsterdam preoperative anxiety and information scale, *NRS* numeric rating scale. ^a^ Models are adjusted for age, gender, education, number of previous surgeries, and grade of procedure. ^b^ Reference category: No desire for anxiolytic medication. ^c^ Models included both anxiety dimensions simultaneously to examine whether anesthesia anxiety and surgery anxiety have unique associations with the desire for anxiolytic medication.
