# Supplementary material for: Patients’ desires for anxiolytic premedication – an observational study in adults undergoing elective surgery
Source: BMC Psychiatry. 2022 Mar 17;22:193. doi: 10.1186/s12888-022-03845-y (PMC8932104; doi:10.1186/s12888-022-03845-y)
Supplement: Supplementary file 4 — Additional file 4: Supplementary Figure 1. ROC curve. Description: Receiver operating characteristic (ROC) curve of prediction of desire for preoperative anxiolytic medication (premedication) using total anxiety scores. [file 12888_2022_3845_MOESM4_ESM.docx]

**Additional file 4** – ROC curve

Supplementary Figure 1 - Receiver operating characteristic (ROC) curve of prediction of desire for preoperative anxiolytic medication (premedication) using total anxiety scores


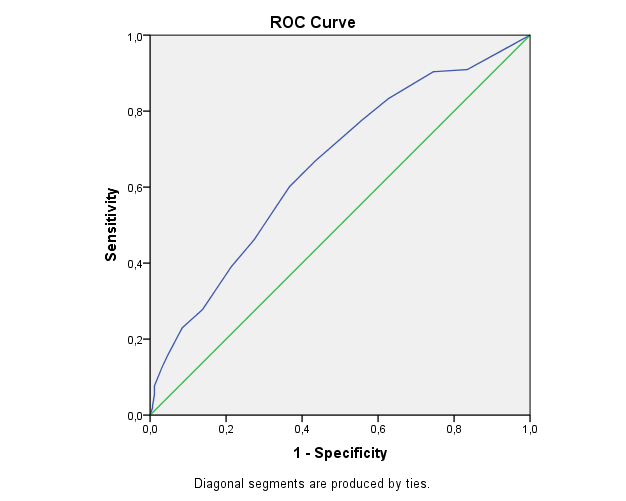


Receiver operating characteristic (ROC) curve plots the true positive rate (sensitivity) against the false positive rate (1-specifity) and displays which total anesthesia and surgery anxiety (APAIS-A-T) cut-off score would best predict who would welcome anxiolytic medication (yes vs. no and on request).
