# Supplementary material for: Patients’ desires for anxiolytic premedication – an observational study in adults undergoing elective surgery
Source: BMC Psychiatry. 2022 Mar 17;22:193. doi: 10.1186/s12888-022-03845-y (PMC8932104; doi:10.1186/s12888-022-03845-y)
Supplement: Supplementary file 5 — Additional file 5: Supplementary Table 6. Crosstab of desire for anxiolytic medication (no/yes/on request) depending on total anesthesia and surgery anxiety (APAIS-A-T) level in all patients. [file 12888_2022_3845_MOESM5_ESM.docx]

**Additional file 5 -** Anxiety level and desire for anxiolytic medication in all patients

Supplementary Table 6

|  | Desire for anxiolytic medication | | |  |
| --- | --- | --- | --- | --- |
| APAIS-A-T | No | Yes | On request | Σ |
| 4 | 90 | 32 | 12 | 134 |
| 5 | 39 | 2 | 16 | 57 |
| 6 | 55 | 25 | 18 | 98 |
| 7 | 27 | 20 | 16 | 63 |
| 8 | 47 | 38 | 29 | 114 |
| 9 | 25 | 24 | 17 | 66 |
| 10 | 33 | 49 | 24 | 106 |
| 11 | 16 | 25 | 21 | 62 |
| 12 | 24 | 40 | 23 | 87 |
| 13 | 11 | 17 | 22 | 50 |
| 14 | 7 | 26 | 17 | 50 |
| 15 | 2 | 11 | 7 | 20 |
| 16 | 6 | 17 | 6 | 29 |
| 17 | 0 | 8 | 0 | 8 |
| 18 | 0 | 10 | 3 | 13 |
| 19 | 0 | 2 | 0 | 2 |
| 20 | 2 | 7 | 2 | 11 |
| Σ | 384 | 353 | 233 | 970 |

*APAIS* Amsterdam preoperative anxiety and information scale, *APAIS-A-T* APAIS anxiety about anesthesia and surgery score (total APAIS anxiety score).
