# Supplementary material for: Patients’ desires for anxiolytic premedication – an observational study in adults undergoing elective surgery
Source: BMC Psychiatry. 2022 Mar 17;22:193. doi: 10.1186/s12888-022-03845-y (PMC8932104; doi:10.1186/s12888-022-03845-y)
Supplement: Supplementary file 6 — Additional file 6: Supplementary Table 7. Crosstab of desire for anxiolytic medication (no/yes/on request) depending on total anesthesia and surgery anxiety (APAIS-A-T) level in anxious patients (no/yes). [file 12888_2022_3845_MOESM6_ESM.docx]

**Additional file 6** Anxiety level and desire for anxiolytic medication in anxious patients

Supplementary Table 7

|  | Desire for anxiolytic medication | | |  |
| --- | --- | --- | --- | --- |
| APAIS-A-T | No | Yes | On request | Σ |
| 4 | 1 | 2 | 0 | 3 |
| 5 | 2 | 1 | 4 | 7 |
| 6 | 6 | 7 | 1 | 14 |
| 7 | 5 | 5 | 4 | 14 |
| 8 | 11 | 15 | 11 | 37 |
| 9 | 11 | 12 | 10 | 33 |
| 10 | 23 | 34 | 17 | 74 |
| 11 | 12 | 20 | 16 | 48 |
| 12 | 19 | 33 | 22 | 74 |
| 13 | 9 | 16 | 21 | 46 |
| 14 | 4 | 26 | 16 | 46 |
| 15 | 2 | 11 | 7 | 20 |
| 16 | 6 | 17 | 6 | 29 |
| 17 | 0 | 8 | 0 | 8 |
| 18 | 0 | 10 | 3 | 13 |
| 19 | 0 | 2 | 0 | 2 |
| 20 | 2 | 7 | 2 | 11 |
| Σ | 113 | 226 | 140 | 479 |

*APAIS* Amsterdam preoperative anxiety and information scale, *APAIS-A-T* APAIS anxiety about anesthesia and surgery score (total APAIS anxiety score).
