# Supplementary material for: Patients’ desires for anxiolytic premedication – an observational study in adults undergoing elective surgery
Source: BMC Psychiatry. 2022 Mar 17;22:193. doi: 10.1186/s12888-022-03845-y (PMC8932104; doi:10.1186/s12888-022-03845-y)
Supplement: Supplementary file 7 — Additional file 7: Supplementary Table 8. Crosstab of desire for anxiolytic medication (no/yes/on request) depending on total anesthesia and surgery anxiety (APAIS-A-T) level in non-anxious patients (no/yes). [file 12888_2022_3845_MOESM7_ESM.docx]

**Additional file 7** Anxiety level and desire for anxiolytic medication in non-anxious patients

Supplementary Table 8

|  | Desire for anxiolytic medication | | |  |
| --- | --- | --- | --- | --- |
| APAIS-A-T | No | Yes | On request | Σ |
| 4 | 89 | 30 | 12 | 131 |
| 5 | 37 | 1 | 12 | 50 |
| 6 | 49 | 18 | 17 | 84 |
| 7 | 22 | 15 | 12 | 49 |
| 8 | 36 | 23 | 18 | 77 |
| 9 | 14 | 12 | 7 | 33 |
| 10 | 10 | 15 | 7 | 32 |
| 11 | 4 | 5 | 5 | 14 |
| 12 | 5 | 7 | 1 | 13 |
| 13 | 2 | 1 | 1 | 4 |
| 14 | 3 | 0 | 1 | 4 |
| 15 | 0 | 0 | 0 | 0 |
| 16 | 0 | 0 | 0 | 0 |
| 17 | 0 | 0 | 0 | 0 |
| 18 | 0 | 0 | 0 | 0 |
| 19 | 0 | 0 | 0 | 0 |
| 20 | 0 | 0 | 0 | 0 |
| Σ | 271 | 127 | 93 | 491 |

*APAIS* Amsterdam preoperative anxiety and information scale, *APAIS-A-T* APAIS anxiety about anesthesia and surgery score (total APAIS anxiety score).
